# Angioscopic Evaluation of In-Stent Occlusion in the Superficial Femoral Artery Treated With Excimer Laser Atherectomy

**DOI:** 10.1016/j.jaccas.2025.103522

**Published:** 2025-06-04

**Authors:** Munehiro Iiya, Yuko Onishi, Ryota Arai, Kosuke Nakada, Hiroko Okazawa, Hiroshi Yoshikawa, Yoshihiro Hanyu, Isshi Kobayashi, Taishi Yonetsu, Tetsuo Sasano

**Affiliations:** a Department of Cardiology, Hiratsuka Kyosai Hospital, Hiratsuka, Japan; b Institute of Science Tokyo Hospital, Tokyo, Japan

**Keywords:** angioscopy, endovascular therapy, excimer laser atherectomy, in-stent occlusion

## Abstract

**Background:**

In-stent occlusion (ISO) comprises various components, including thrombus and neointimal hyperplasia, but its detailed characteristics and treatment responses remain unclear.

**Case Summary:**

We performed excimer laser atherectomy (ELA) to treat an ISO in the superficial femoral artery. Multimodal imaging, including intravascular ultrasound, optical coherence tomography, and angioscopy, enabled detailed procedural assessment. Additionally, real-time angioscopy was performed from a contralateral approach during the ELA procedure to observe its effects dynamically. The results showed that although ELA had a limited effect on vaporizing the proximal cap and neointimal hyperplasia, it successfully dissipated thrombus and inflammatory tissue.

**Discussion:**

This is the first report to evaluate the effects of ELA on ISO lesions using multimodal imaging, including real-time angioscopy. This approach provides new insights into the potential benefits of ELA in endovascular therapy.

**Take-Home Message:**

ISO contains various components, including thrombus and neointimal hyperplasia. ELA demonstrated differential effects on each component.

## Graphical Abstract

**Figure:**
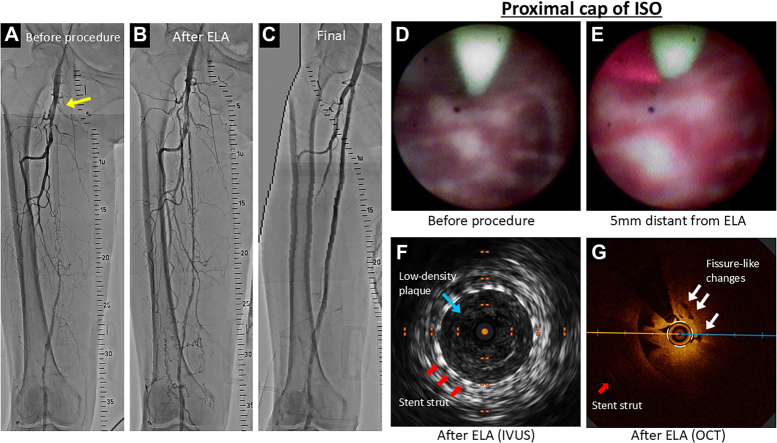

## History of Presentation

An 81-year-old woman with chronic total occlusion (CTO) in the right superficial femoral artery (SFA) underwent deployment of a bare-metal stent (S.M.A.R.T. 5.0 × 20 mm, Cordis) to relieve leg claudication. However, her ankle-brachial index decreased again, and symptoms worsened within 10 months. Thirteen months after stent placement, angiography revealed an in-stent occlusion (ISO) of the proximal SFA. Given her worsening symptoms, classified as Rutherford category 3 claudication, she opted for percutaneous revascularization 14 months after initial stent implantation.

**Take-Home Messages**

• ISO contains various components, including thrombus and neointimal hyperplasia.
• ELA demonstrated differential effects on each component.

## Past Medical History

The patient had a medical history that included hypertension, bronchial asthma, CTO of the right SFA, and no prior cardiovascular events other than the initial stent placement.

## Differential Diagnosis

The differential diagnosis for the ISO included stent restenosis with thrombus, neointimal hyperplasia, and mixed components within the lesion.

## Investigations

**Figure 1:**
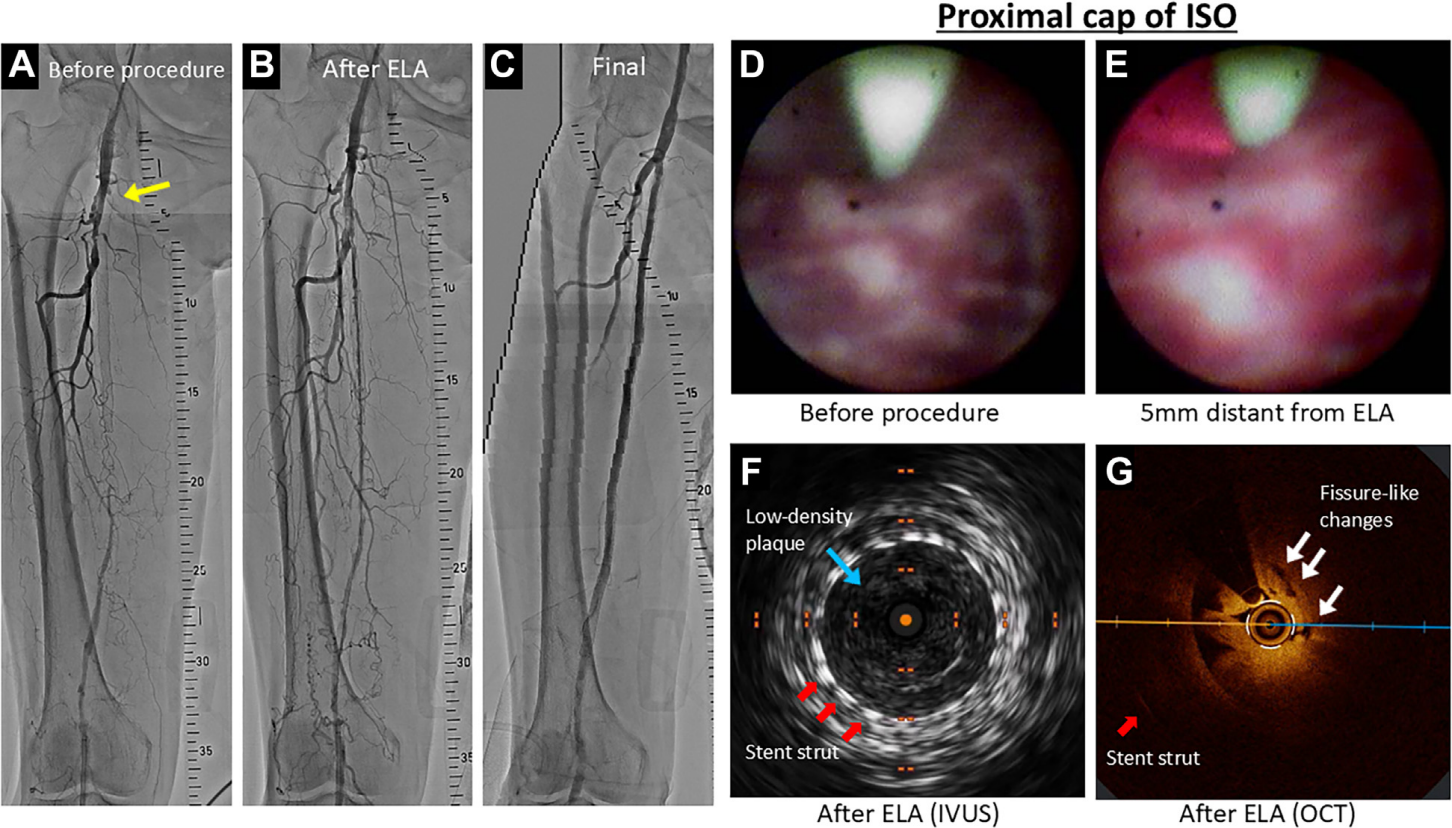
Angiogram, OCT, IVUS, and Angioscopy During Procedure (A) The initial angiogram confirmed an in-stent occlusion (ISO) of the proximal segment of the superficial femoral artery (SFA) stent, with an abrupt ostium (yellow arrows). (B) After excimer laser atherectomy (ELA) was performed on the culprit lesion, angiography revealed successful recanalization of the SFA. (C) The final angiogram demonstrated a well-dilated SFA with adequate flow. (D) Initial angioscopy revealed heterogeneous proximal cap components including white neointimal hyperplasia and some inflammatory tissues with red color. (E) At 5 mm distant from the ELA catheter, red light emitted by the laser was visible through the angioscope; however, there was no apparent modification of the proximal cap. (F) IVUS after ELA demonstrated that the proximal cap of the ISO consisted of low-density plaques (blue arrows), suggesting relatively soft plaques. (G) After ELA, optical coherence tomography (OCT) at the proximal cap of the lesion revealed attenuated plaques with a protruding morphology accompanied by several small fissure-like changes (white arrow).

A 6-F guiding sheath was inserted from the left common femoral artery and advanced to the right common femoral artery via the iliac arteries. The initial angiogram confirmed an ISO of the proximal segment of the SFA stent, with an abrupt ostium (Figure 1A, Video 1 [arrow]).

## Management (Medical/Interventions)

To enable a bidirectional approach, another 6-F sheath was inserted to the right popliteal artery. A 0.014-inch guidewire (CROSSLEAD Tracker, Asahi Intecc) along with a microcatheter (Ichibanyari, Kaneka Medical Products) were advanced antegradely and successfully entered through the proximal cap of the culprit lesion. The wire continued to advance and passed through the healthy popliteal artery. The guidewire was further advanced into the popliteal sheath, allowing externalization.

**Figure 2:**
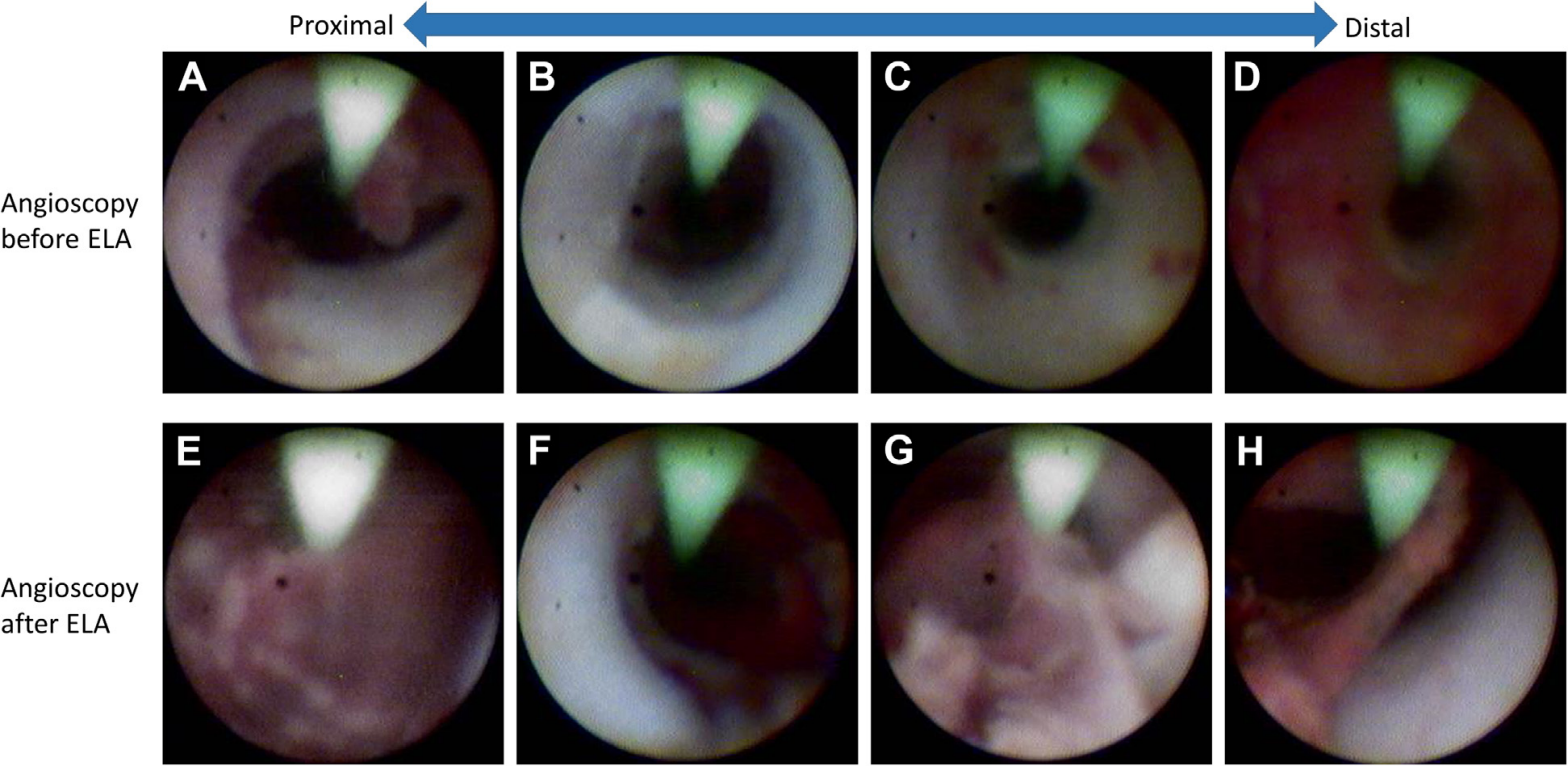
Comparison of Angioscopy Before and After ELA (A to D) Initial angioscopy performed via the retrograde approach revealed white neointimal hyperplasia, which seemed to be relatively tough tissues, accompanied by diffuse red thrombus and inflammatory tissue components. (E to H) Angioscopy was performed via the antegrade approach after excimer laser atherectomy (ELA). While the previously noted white neointimal hyperplasia was fragmented and diffusely floating in a patchy pattern, red thrombus and inflammatory tissue initially observed were decreased.

Angioscopy performed via the retrograde approach revealed white neointimal hyperplasia, which seemed to be relatively tough tissue, accompanied by diffuse red thrombus and inflammatory tissue components (Video 2, Figures 2A to 2D).

Excimer laser atherectomy (ELA) was then performed using a Turbo Elite 2.0-mm catheter (Philips) retrogradely, while angioscopic observation was performed simultaneously via the antegrade approach using the same guidewire during the laser procedure. This unique approach allowed real-time visualization of the lesion during treatment, enabling continuous monitoring of the effects of ELA on the target tissue. ELA was performed with output flow range of 45 mJ/mm^2^ and a repetition rate of 25 pulses/s. The laser catheter was advanced slowly during activation. The excimer laser was applied in intervals of up to 20 seconds per activation period.

On angioscopy, no significant changes were observed in the proximal cap of the lesion, located 10 to 20 mm from the ELA catheter. At 5 mm distant from the ELA catheter, red light emitted by the laser was visible through the angioscope; however, there was no apparent modification of the proximal cap (Video 3, Figures 1D and 1E). After removing the angioscope, the ELA catheter successfully crossed the proximal cap. Once the cap was crossed, ablation was continued as the catheter was slowly withdrawn back through the distal SFA.

After performing ELA on the culprit lesion, angiography revealed successful recanalization of the SFA (Figure 1B, Video 4). Angioscopy revealed that the previously noted white neointimal hyperplasia was fragmented and diffusely floating in a patchy pattern. Rather than complete vaporization, the ELA appeared to mechanically disrupt these relatively steady tissues, tearing them into smaller pieces. Furthermore, red thrombus and inflammatory tissue initially observed were decreased, suggesting that these components had been ablated or diminished through the ELA procedure (Video 5, Figures 2E to 2H).

Intravascular ultrasound (IVUS) demonstrated that the culprit lesions including proximal cap of the ISO were diffuse low-density plaques, suggesting relatively soft plaques or neointimal hyperplasia (Video 6, Figure 1F). Optical coherence tomography (OCT) revealed attenuated plaques extending throughout the lesion, with a protruding morphology observed at the proximal cap of the lesion. Furthermore, several small fissure-like changes were identified at the proximal cap, which may represent modifications induced by the ELA procedure (Video 7, Figure 1G).

Dilation using a 5.0-mm balloon (Starling 5.0 × 220 mm, Boston Scientific) was performed, but the proximal cap showed insufficient dilatation, reflecting the relatively tough nature of the tissue. Additional dilation was performed using a 6.0 × 20 mm cutting balloon (Boston Scientific), which successfully improved the dilation of the proximal cap. Subsequently, a S.M.A.R.T. stent (6.0 × 150 mm) was deployed in the distal SFA, followed by postdilation using a 6.0-mm balloon (Metacross 6.0 ×60 mm, Terumo). Drug-coated balloons (IN.PACT 6.0 × 120 mm and 6.0 × 150 mm, Medtronic) were inflated in the ISO and distal SFA, respectively. The final angiogram demonstrated well dilated SFA with adequate flow (Figure 1C, Video 8).

## Outcome and Follow-Up

The patient showed significant alleviation of symptoms, with normalization of the ankle-brachial index and no recurrence of claudication. Follow-up duplex ultrasound performed 3 months after the procedure confirmed continued patency of the treated segment, without evidence of restenosis.

## Discussion

The present case highlights the heterogeneous components constituting ISO of the SFA and the different reaction to ELA of each component. Using multimodal imaging (angioscopy, IVUS, and OCT), we observed that ELA had a limited effect on the proximal cap of the ISO and white neointimal hyperplasia, disrupting these steady tissues and tearing them into smaller pieces. In contrast, ELA effectively vaporized soft components, such as red thrombus and inflammatory tissues.

Real-time angioscopic findings during the ELA procedure indicated that the photoablation effect of ELA was limited to a short range, particularly in stable tissues such as the proximal cap of the ISO as observed in the present case. This implies that ELA’s ablative power diminishes with distance, thereby limiting its penetration depth.

In contrast, a previous case reported that ELA caused perforation of an ISO lesion.^1^ In our case, the perforation might have occurred when ELA was performed with more mechanical push force to overcome the index tissue along with the photoablation itself. Thus, we suggest that ELA be performed with maximum attention to the resistance from the device so as not to overcome inappropriate power to pass the lesion.

This case also demonstrates that ISO lesions contain heterogeneous components, which is in line with previous findings.^2^^,^^3^ Through angioscopic evaluation after ELA, we confirmed differential reactions to the device among thrombus, hyperplasia, and inflammatory tissue (Figures 2A to 2H, Videos 2 and 5).

The CTO proximal cap has been reported to be composed of stable tissue that is often refractory to endovascular therapy.^4^^,^^5^ In the present case, multimodal imaging with IVUS and OCT provided further characterization. Although angioscopy revealed that the proximal cap consisted of white hyperplasia, reflecting a limited response to balloon inflation, IVUS demonstrated low-attenuation plaques in the same region, suggesting the presence of relatively soft components. OCT also revealed attenuated tissue within the proximal cap and indicated ELA-induced modifications, represented by small fissure-like changes. Further evaluation were needed to confirm these findings.

## Conclusions

Angioscopic evaluation of SFA CTO lesions treated with ELA is rare, and the present real-time evaluation of CTO proximal cap is novel. In addition, multimodal imaging including IVUS and OCT indicated different responses to ELA within the CTO lesion.

## Funding Support and Author Disclosures

The authors have reported that they have no relationships relevant to the contents of this paper to disclose.

## Appendix

Video 1 Angioscopy After Wiring

Video 2 Angioscopy During ELA

Video 3 Angioscopy After ELA

Video 4 IVUS After ELA

Video 5 OCT After ELA

Video 6 Initial Angiogram

Video 7 Angiogram After ELA

Video 8 Final Angiogram
